# Turnover rate of coenzyme A in mouse brain and liver

**DOI:** 10.1371/journal.pone.0251981

**Published:** 2021-05-21

**Authors:** Laura Orsatti, Maria Vittoria Orsale, Pamela di Pasquale, Andrea Vecchi, Fabrizio Colaceci, Alina Ciammaichella, Ilaria Rossetti, Fabio Bonelli, Karsten Baumgaertel, Kai Liu, Daniel Elbaum, Edith Monteagudo

**Affiliations:** 1 ADME/DMPK Department, IRBM SpA, Pomezia, Roma, Italy; 2 Medicinal Chemistry Department, IRBM SpA, Pomezia, Roma, Italy; 3 Retrophin, San Diego, CA, United States of America; University of Cordoba, SPAIN

## Abstract

Coenzyme A (CoA) is a fundamental cofactor involved in a number of important biochemical reactions in the cell. Altered CoA metabolism results in severe conditions such as pantothenate kinase-associated neurodegeneration (PKAN) in which a reduction of the activity of pantothenate kinase isoform 2 (PANK2) present in CoA biosynthesis in the brain consequently lowers the level of CoA in this organ. In order to develop a new drug aimed at restoring the sufficient amount of CoA in the brain of PKAN patients, we looked at its turnover. We report here the results of two experiments that enabled us to measure the half-life of pantothenic acid, free CoA (CoASH) and acetylCoA in the brains and livers of male and female C57BL/6N mice, and total CoA in the brains of male mice. We administered (intrastriatally or orally) a single dose of a [^13^C_3_-^15^N-^18^O]-labelled coenzyme A precursor (fosmetpantotenate or [^13^C_3_-^15^N]-pantothenic acid) to the mice and measured, by liquid chromatography-mass spectrometry, unlabelled- and labelled-coenzyme A species appearance and disappearance over time. We found that the turnover of all metabolites was faster in the liver than in the brain in both genders with no evident gender difference observed. In the oral study, the CoASH half-life was: 69 ± 5 h (male) and 82 ± 6 h (female) in the liver; 136 ± 14 h (male) and 144 ± 12 h (female) in the brain. AcetylCoA half-life was 74 ± 9 h (male) and 71 ± 7 h (female) in the liver; 117 ± 13 h (male) and 158 ± 23 (female) in the brain. These results were in accordance with the corresponding values obtained after intrastriatal infusion of labelled-fosmetpantotenate (CoASH 124 ± 13 h, acetylCoA 117 ± 11 and total CoA 144 ± 17 in male brain).

## Introduction

Coenzyme A (CoA) has a clearly defined role as a cofactor for a number of oxidative and biosynthetic reactions in intermediary metabolism [1,2]. It is involved in cellular metabolism (fatty acid synthesis, tricarboxylic acid cycle, oxidation of fatty acid, bile acid conjugation), production of neurotransmitters, regulation of gene expression [3], redox regulation, protein quality control and autophagy [4,5]. In the cell, CoA is present as unesterified (CoASH) and as esterified (acylCoA) forms. AcylCoAs are grouped into short-, medium- and long-chain esters based on the length of the carbon chain group forming a thioester linkage with CoA. Their level and distribution vary among organs, and their cellular localization (mainly in the cytosol, mitochondria and peroxisomes) is tightly regulated to support specific metabolic pathways. To perform its multiple functions, CoA levels may change according to the physiology of the cellular environment of the different tissues and organs. The cellular and the tissue distribution of CoA levels depend on the localization and expression in the different tissues and organs of the enzymes involved in its biosynthesis and catabolism [6–10]. Its steady state tissue level is the result of the relative rate of biosynthesis and turnover. CoA biosynthesis occurs via a highly conserved pathway that involves five enzymatic steps and utilizes pantothenate (vitamin B5), adenosine triphosphate and cysteine. In most known organisms, pantothenate is easily obtained from a normal diet, nevertheless, bacteria, yeast and plants can synthesize pantothenate from β-alanine. Five enzymes are responsible for the intracellular CoA biosynthesis: pantothenate kinase (PANK; EC 2.7.1.33), 4’-phosphopantothenoylcysteine synthetase (PPCS; EC 6.3.2.5), (*R*)-4’-phospho-N-pantothenoylcysteine decarboxylase (PPCDC; EC 4.1.1.36), 4’-phosphopantetheine adenylyltransferase (PPAT; EC 2.7.7.3) and dephospho-CoA kinase (DPCK; EC 2.7.1.24). The phosphorylation of pantothenate by PANK is the first and rate-controlling step for CoA biosynthesis. The final two steps (i.e. adenylyltransferase and kinase activity) in eukaryotes and mammals are performed by a single bifunctional enzyme, called CoA synthase (COASY) [11]. While the pathway of CoA biosynthesis has been fully characterized, the CoA degradation pathway is yet to be fully elucidated. Several enzymes are involved in this process, including lysosomal acid phosphatases [12], nudix hydrolases [13–16], nucleotide pyrophosphatases [17], and pantetheinases [18]. The first three enzymes lead to the formation of pantetheine, which is processed by pantetheinases to create pantothenic acid (PA) and cysteamine. Defective CoA biosynthesis and homeostasis have been associated with various diseases including diabetes [19,20], Reye’s syndrome, cancer [21], neurodegeneration [22], pantothenate kinase-associated neurodegeneration (PKAN) [23], vitamin B12 deficiency [24] and cardiac hypertrophy [25–28]. In particular, PKAN is a genetic movement disorder caused by mutations in the human *PANK2* gene coding for pantothenate kinase 2 (PANK2), a PANK isoform highly expressed in the brain in human neuronal tissues [23,29]. Mutations in *PANK2* gene give rise to the expression of a PANK protein with little or no catalytic activity, causing a severe, rare neurodegenerative disease associated with abnormal iron accumulation in the brain, and characterized by progressive dystonia and Parkinsonism. Currently, there are no therapies available to treat PKAN. Few approaches have been explored so far, including the use of protected phosphopantothenates (for example, fosmetpantotenate) [30,31] or PKAN modulators to reverse hopantenate inhibition of CoA synthesis [32,33].

The successful use of the previously mentioned agents to increase CoA in the CNS requires a complex interplay between bio-distribution and metabolism. Adequate delivery of the intact prodrug to the systemic circulation and consequent distribution across the blood-brain barrier must ultimately be balanced by the incorporation of sufficiently labile functionality (such that the bioactive species, phosphopantothenic acid can be released in target tissue).

A novel approach to PKAN therapy has been proposed recently by Sharma LK *et al*. [34] consisting in the activation of alternate PANK isoforms. The authors developed an allosteric PANK3 activator that crosses the blood brain barrier and is able to increase CoA levels in mouse brain after oral administration to a knockout mouse model of brain CoA deficiency.

In the prospective of developing a therapy to treat PKAN or other neurodegenerative diseases linked to CoA deficiency in brain, one important piece of information that is currently lacking is the CoA turnover rate in the target organ. This information is crucial to design novel disease treatments aimed to restore and maintain physiologically relevant levels of CoA. CoA half-life would directly impact the frequency of therapeutic dosing.

To address this knowledge gap, here we report the determination of the half-lives of CoASH, acetylCoA and total CoA *in vivo* in the brains and livers of mice. Formation and disappearance over time of endogenous CoA biosynthetically labelled with ^13^C and ^15^N was measured using liquid chromatography-mass spectrometry (LC-MS). Half-life was determined based on the rate of decay of the labelled CoA species.

## Methods

### Chemicals

[^13^C_3_-^15^N]-PA-labelled ((*R*)-3-(2,4-dihydroxy-3,3-dimethylbutanamido)propanoic acid) was synthesized in house. (^18^O, ^15^N, ^13^C_3_)-fosmetpantotenate +6 atomic mass unit (AMU) [29] was synthesized in house, purity 99.9% by UHPLC, distereomer ratio 54/48 from ^31^P NMR. Calcium pantothenate was supplied by EDQM (Strasbourg, France). CoASH was purchased from Larodan AB (SOLNA, Sweden). AcetylCoA Na^+^ salt, atenalol and KOH were obtained from Sigma-Aldrich (MO, USA). HPLC-MS grade ACN, water, TFA and formic acid (FA) were obtained from Fluka.

### Preparation of PA, CoASH and acetylCoA stock and working solutions

PA, CoASH and acetylCoA stock solutions were freshly prepared at 10 mM in 50 mM ammonium acetate pH 6.0. Working solutions (WS) were prepared in the same buffer by serial dilutions of the stock. Atenolol (internal standard (IS) for tissue samples) stock solution was prepared in DMSO at 1 mg/mL and stored for 2 months at -20°C. Working solution for IS (WS-IS) was freshly prepared before use at 2.5 μg/mL in methanol (MeOH)/50 mM ammonium acetate pH 6.0 70/30 (v/v) for PA, CoASH and acetylCoA determination and in 50 mM ammonium acetate pH 6.0 for total CoA determination.

### Animal studies

#### Oral (PO) administration study in C57BL/6N mice

This study was conducted at IRBM in full compliance with the EU Directive 63/2010 (On the Protection of Animals Used for Scientific Purposes) and its Italian transposition (Italian Decree no. 26/2014) as well as with all applicable Italian legislation and relevant guidelines.

IRBM is authorized by the Italian Ministry of Health and local veterinary authority to house, breed, and use laboratory rodents for scientific purposes. Protocol was approved by the internal IRBM Ethics Committee and by the Italian Ministry of Health. Male and female C57BL/6N mice (5 weeks old, Charles River (Como, Italy)) were housed in individual ventilated cages (IVCs Tecniplast) with sawdust as bedding. Cages were identified by a color code label recording the sample ID, animal number and details of treatment (route, dose and time point). Animals were identified with a unique number on each tail via permanent markers. Room temperature was maintained at approximately 22°C with relative humidity between 40–70% and an average daily airflow of at least 10 fresh air changes per hour. Rooms were lit by fluorescent tubes controlled to give an artificial cycle of 12 h light and 12 h dark each day.

All animals were monitored two times a week and clinical signs were recorded. The monitored parameters included changes in the skin, eyes, nose, mouth, head, breath, urines, feci and in locomotor activity. Animals were also weighed two times a week to monitor the body mass change. In case of suffering or more than 20% loss of body weight animals were euthanized.

In a preliminary study conducted in male mice, a low PA diet period of three and six days before treatment was evaluated to facilitate the incorporation of [^13^C_3_-^15^N]-PA into CoASH and acetylCoA. Then, levels of endogenous PA, CoASH and acetylCoA were measured. For three and six days animals (n = 3) were fed with PA deficient diet (Mucedola MD 95248). Brain and liver samples were collected at day 0, 3 and 6. The mice were anesthetized using Isoflurane. The sacrifice was performed in accordance with IRBM standard operation procedure and in compliance with EU/Italian laws (decapitation/dislocation in a state of anaesthesia). Immediately after the mouse sacrifice, the whole brains and livers were explanted, washed with refrigerated saline solution (4°C), and divided into two halves, then weighed and snap frozen in dry ice in Precellys® (Bertin Technologies, Montigny-le-Bretonneux, France) tubes. Brains and livers were then stored at -80°C until LC-MSMS analysis.

In the [^13^C_3_-^15^N]-PA PO administration study for 3 days all animals were fed with PA deficient diet (Mucedola MD 95248). Then, just before oral administration of [^13^C_3_-^15^N]-PA, standard rodent diet (Mucedola 4RF21) was administered until the end of the study. Animals were offered drinking water *ad libitum* (Mucedola, Milano, Italy). All animals were weighed immediately before testing. PA-free food intake was measured daily and the consumption was calculated. Animals (approximately 6 weeks old) were dosed orally in a fed state. The appropriate dose volume of [^13^C_3_-^15^N]-PA, calculated per each individual animal according to body weight (administration volume: 10 mL/kg), was administered orally using a gastric cannula connected to a 300 μL syringe (BD Plastipak 3/10 cc insulin syringe U-100 28g cat n. 309300). Liver and tissue samples were collected at the following time points: pre-dose, 6, 18, 30, 54, 78, 102, 150, 198, 246, 294, and 342 h post-dosing and treated as described for the preliminary low PA study. Animals were well for all the duration of the study. No sign of suffering was observed.

#### Intrastriatal administration study in C57/Bl6 mice

The *in vivo* study was conducted at Charles River Labs in accordance with protocols approved by the Institutional Animal Care and Use Committee of Charles River Laboratories, SSF. Twenty-seven male C57/Bl6 mice (n = 27, CRL, 8 weeks old) were used for the experiment. Upon arrival, animals were group-housed (n = 2-5/cage) with access to food and water *ad libitum*. Animals were maintained on a 12/12 h light/dark cycle (lights ON at 7:00 AM) in a temperature- (22 ± 2°C) and humidity (approx. 50%) controlled room. Animals were acclimated for at least 7 days prior to the beginning of the experiment.

Three mice were not dosed (time 0 group) while 24 mice received bilaterally infusion with fosmetpantotenate (+6 AMU) into the dorsal striatum (5 μL/side, 25 μg/μL, total dose 250 μg). To do so, mice were anesthetized using Isoflurane (2%, 800 mL/min O_2_). Bupivacaine was used for local analgesia and carprofen was used for peri-/post-operative analgesia. The animals were placed in a stereotaxic frame (Kopf instruments, USA). The following coordinates were used for the dorsal striatum (antero-posterior: +0.8 mm and lateral: ±1.4 from bregma, dorso-ventral: -1.7 mm from dura). Utilizing a Hamilton syringe (model # 80308; 10 μL syringe with corresponding 30 ga blunt tip needle) and the stereotactic micromanipulator, the location of the bur holes were designated and drilled. The infusion cannula was lowered into the brain to the depth of the desired location and infusion conducted at a rate of 0.5 μL/min. When delivery was completed, a 5-minute waiting period was applied before withdrawing the needle. Following the waiting period, the contralateral side was infused in the same manner. Once the two infusions were completed, the skin incision was closed with sutures. At the appropriate takedown time (0, 24, 48, 96, 144, 192, 240, 288, or 336 h after treatment infusion), mice (n = 3) were euthanized via CO_2_. Brain tissue was extracted, separated into left and right hemisphere, immediately frozen (-80°C) in separate Precellys® tubes and sent to IRBM for analysis.

### Mouse tissue sample preparation

#### Determination of unlabelled and labelled PA, CoASH and acetylCoA

Tissue samples (brain and liver) were thawed in an ice bath for 15 min. Brain hemispheres (or half liver) were homogenized with 4 volumes of MeOH/50 mM ammonium acetate pH 6.0 70/30 (v/v) containing 31.2 ng/mL (brain) or 125 ng/mL (liver) atenolol as internal standard using a Precellys® 24 homogenizer at 5000 rpm for 15 s. After vortex and centrifugation (16,000 rcf, 15 min at 4°C) supernatants were either dried under nitrogen and reconstituted with 50 mM ammonium acetate pH 6.0 buffer or diluted with the same buffer and directly analysed by LC-MS.

#### Determination of unlabelled and labelled total CoA

Brain tissue samples were thawed in an ice bath for 15 min, 4 volumes of aqueous 0.25 M potassium hydroxide solution were added and homogenized with Precellys® 24 homogenizer (5,000 rpm, 15 s). Then, the extracts were heated at 55°C and after 10 min an aliquot of a 10–50% acetic acid solution was added to each sample to adjust the pH to 5.0. Samples were then centrifuged at 14,000 rcf for 15 min at 4°C and supernatants were diluted with 50 mM ammonium acetate pH 6.0 containing the internal standard (2.5 μg/mL atenolol) and analyzed by LC-MSMS.

### LC-MS analysis

#### LC-HRMS method for simultaneous quantitation of unlabelled and labelled (+4 AMU) PA, CoASH and acetylCoA (PO study)

An Ultimate 3000 UHPLC system coupled with an Orbitrap QExactive™ mass spectrometer (Thermo Scientific) was used for the analysis of brain and liver extracts of the PO study. Chromatographic separation was performed with a Merck Chromolith Performance C18 column (2.0 x 100 mm). The mobile phases were A) 2.5 mM ammonium acetate pH 6.7 and B) acetonitrile (ACN)/2-propanol 98:2 (v/v). The eluting gradient was as follows: the column was equilibrated with 1% B for 0.25 min, B increased to 70% in 2.15 min, then further increased to 90% in 0.60 min, remained constant for 0.50 min, decreased to 1% in 0.10 min and remained 1% for 3.40 min. The flow rate was 0.3 mL/min and the column T 25°C. The injection volume was 2 μL.

For MS analysis the following parameters were used: ion spray voltage 3.2 kV, sheath gas flow 50 a.u., aux gas flow 5 a.u., aux T 300°C, capillary T 320°C, S-lens RF level 50%. Full MS parameters were as follows: mass range 150–2000, AGC target 1x10^6^, max injection time 64 msec, resolution 35,000 (FWHM @ 200 *m/z*). For targeted SIM (tSIM) analysis the following settings were used: isolation window 1 Da, AGC target 1x10^6^, resolution 140,000 (FWHM @ 200 *m/z*). For PA tSIM settings were: *m/z* 220.1179 (PA), 224.1250 (+4 AMU labelled PA), acquisition range 1.2–1.7 min, MSX count 2, max injection time 128 msec. For CoASH tSIM settings were: *m/z* 768.1225 (CoASH), 772.1296 (+4 AMU labelled CoASH), acquisition range 1.7–2.0 min, MSX count 3, max injection time 128 msec. For atenolol tSIM settings were: *m/z* 267.1701, acquisition range 1.9–2.3 min, MSX count 3, max injection time 128 msec.

#### Calibration standards and quality controls

PA, CoASH and acetylCoA (unlabelled and +4 AMU labelled) were simultaneously measured in brain and liver extracts. Calibration standards (CSs) and quality controls (QCs) were prepared in matrix (brain and liver homogenate supernatants of pre-dose mice) by additions of the unlabelled standard compounds to the tissue extracts. Area ratio (peak area analyte/peak area IS) of endogenous compounds determined in QC0 samples, were then subtracted to CSs and QCs.

For the analysis of the mouse liver, a nine-points standard curve (range 16.6–427 μg/g_liver_ for CoASH, 2.10–53.9 μg/g_liver_ for acetylCoA and 0.190–4.86 μg/g_liver_ for PA) was run at the beginning and at the end of sample analysis and four sets of quality controls in triplicates were included (for CoASH: QC0 = endogenous, QC_low_ = 35.6 μg/g_liver_, QC_med_ = 107.0 μg/g_liver_ and QC_high_ = 320 μg/g_liver_; for acetylCoA: QC0 = endogenous, QC_low_ = 4.50 μg/g_liver_, QC_med_ = 13.5 μg/g_liver_ and QC_high_ = 40.5 μg/g_liver_; for PA: QC0 = endogenous, QC_low_ = 0.406 μg/g_liver_, QC_med_ = 1.22 μg/g_liver_ and QC_high_ = 3.65 μg/g_liver_).

For the analysis of the mouse brain, a nine-points standard curve (range 2.0–51 μg/g_brain_ for CoASH, 0.53–14.0 μg/g_brain_ for acetylCoA and 0.29–7.30 μg/g_brain_ for PA) was run at the beginning and at the end of sample analysis and four sets of quality controls in triplicates were included (for CoASH: QC0 = endogenous, QC_low_ = 4.30 μg/g_brain_, QC_med_ = 13.0 μg/g_brain_ and QC_high_ = 38.0 μg/g_brain_; for acetylCoA: QC0 = endogenous, QC_low_ = 1.10 μg/g_brain_, QC_med_ = 3.40 μg/g_brain_ and QC_high_ = 10.0 μg/g_brain_; for PA: QC0 = endogenous, QC_low_ = 0.61 μg/g_brain_, QC_med_ = 1.80 μg/g_brain_ and QC_high_ = 5.50 μg/g_brain_). For CSs and QCs acceptance criteria was ±30% of accuracy.

For later time point samples in which level of labelled metabolite was below limit of quantification (LOQ), linearity of response between C_max_ and 10% of C_max_ was demonstrated by dilution (2, 5 and 10 folds) of C_max_ sample extract with blank matrix extract (mouse liver and brain). The slope of the so obtained curve was compared to the slope of the unlabelled standard curve. Similar slope (±20%) indicated linearity below LOQ.

#### LC-MSMS method for simultaneous quantitation of unlabelled and labelled (+4 and +6 AMU) CoASH and acetylCoA (intrastriatal study)

An Acquity UPLC I-Class system (Waters Corp., Milford, MA) coupled to an API 6500 triple quadrupole mass spectrometer (AB Sciex, Toronto, Canada) was used for simultaneous quantitation of CoASH and acetylCoA species in mouse brain extracts. The chromatographic conditions used are reported in Table 1.

**Table 1:** Chromatographic conditions for analysis of free CoA (CoASH) and acetylCoA (unlabelled, +4 and +6 AMU) in brain extracts.

****Parameter****	****Setting****	
Instrument	Acquity UPLC I-Class	
Column	Waters HSS T3 C18, 1.7 μm, 2.1 x 50 mm	
Flow rate	0.6 mL/min	
Column T	40°C	
Injection volume	2 μL	
Mobile phase	A: 8.6 mM TEA/100 mM HFIP in H_2_O	
	B: ACN/100 mM TEAA 8/2 (v/v)	
Gradient profile	Time (minutes)	Mobile phase B (%)
	0.00	2
	0.60	2
	1.50	30
	1.80	98
	2.10	98
	2.15	2
	3.80	2

MS analysis was performed using an API 6500 triple quadrupole mass spectrometer with a Turbo IonSpray® ionization source in positive ion mode. Electrospray ionization parameters were set as follows: source temperature, 450°C; curtain gas, 20 psi; gas 1, 40 psi; gas 2, 60 psi; collision gas (CAD) 9; ion spray voltage 5500 V. CoASH and acetylCoA quantitation was accomplished through the use of multiple reaction monitoring (MRM) acquisition methods using the transitions reported in Table 2. Data acquisition and analysis was performed with Analyst™ 1.6.2 (AB Sciex, Toronto, Canada).

**Table 2:** MRM conditions for analysis of free CoA (CoASH) and acetylCoA (unlabelled, +4 and +6 AMU) in brain extracts.

Compound	Q1 (*m/z*)	Q3 (*m/z*)	Dwell Time (ms)	DP	EP	CE	CXP
CoASH (i)	768.0	261.1	60	90	10	41	15
CoASH (ii)	768.0	428.1	30	90	10	36	8
AcetylCoA (i)	809.9	303.1	40	90	10	42	15
AcetylCoA (ii)	809.9	428.1	20	90	10	36	8
Atenolol	267.2	145.0	20	130	7	36	17
CoASH + 4 AMU	772.0	265.1	40	90	10	41	15
CoASH + 6 AMU	774.0	265.1	40	90	10	36	15
AcetylCoA + 4 AMU	813.9	307.1	40	90	10	42	15
AcetylCoA + 6 AMU	815.9	307.1	40	90	10	42	15

DP: Declustering potential; EP: Entrance potential; CE: Collision energy; CXP: Collision cell exit potential.

#### Calibration standards and quality controls

CoASH and acetylCoA (unlabelled, +4 and +6 AMU) were simultaneously measured in brain extracts. CSs and QCs were prepared in C57/Bl6 mouse brain depleted of endogenous CoASH and acetylCoA. The brain was kept at room temperature for 2 days, then homogenised in 4 volumes (w/v) of IS-WS 70/30 MeOH/50 mM ammonium acetate pH 6.0 with a Precellys® 24 homogenizer. The homogenate was centrifuged at 4°C at 16,000 rcf for 15 min and then the supernatant was divided into 40 μL aliquots and spiked with 10 μL of acetylCoA/CoASH spiking solution for CSs and QCs. Supernatant (10 μL) was diluted with 90 μL of 50 mM ammonium acetate pH 6.0 containing 200 ng/mL of atenolol (IS) and directly analysed by LC-MSMS. The standard curve and QCs were for CoASH (range 0.18–23.0 μg/g_brain_, QC0, QC_low_ = 0.90, QC_med_ = 3.6, QC_high_ = 14.0 μg/g_brain_) and for acetylCoA (range 0.046–5.90 μg/g_brain_, QC0, QC_low_ = 0.24, QC_med_ = 0.95, QC_high_ = 3.80 μg/g_brain_). Acceptance criteria was ±30% of accuracy.

### Data analysis and statistics

Calibration curves for quantitation of CoASH and acetylCoA (PO and intrastriatal studies), linearity of response between C_max_ and 10% of C_max_ and area ratio of labelled CoA species versus time plots (semi-log scale) were obtained by weighted (1/X) linear least square regression and were generated using GraphPad Prism version 7.00.

Statistical analysis was performed using GraphPad Prism. Student’s t test was used to calculate p-values. Differences between groups were considered statistically significant when p was <0.02.

### Half-life determination

CoA half-life (t_1/2_) was determined based on the analysis of the disappearance of the labelled-CoA species as a function of time. CoA levels were quantified as the peak area relative to an internal standard (area ratio). Area ratio of labelled species from the C_max_ to the elimination was considered for the half-life calculation. The elimination constant k was calculated by plotting mean area ratio values (n = 3) on a semi-logarithmic scale and fitting with a best fit linear regression. The half-life (t_1/2_) expressed in hours was derived using Eq 1:

$$t_{1/2}=ln2/\left(‐k\right)$$ Eq 1

The associated error (Δt_1/2_) was estimated as the ratio between the error related to the elimination constant and the square of the elimination constant, as indicated in the formula:

$$\Delta t_{1/2}=\Delta k/k^{2}$$ Eq 2

Linear regression, elimination constant and the error related to the elimination constant were generated using GraphPad Prism version 7.00.

## Results

### CoASH and acetylCoA turnover rate in liver and brain of C57BL/6N male and female mice (PO study)

**Fig 1:**
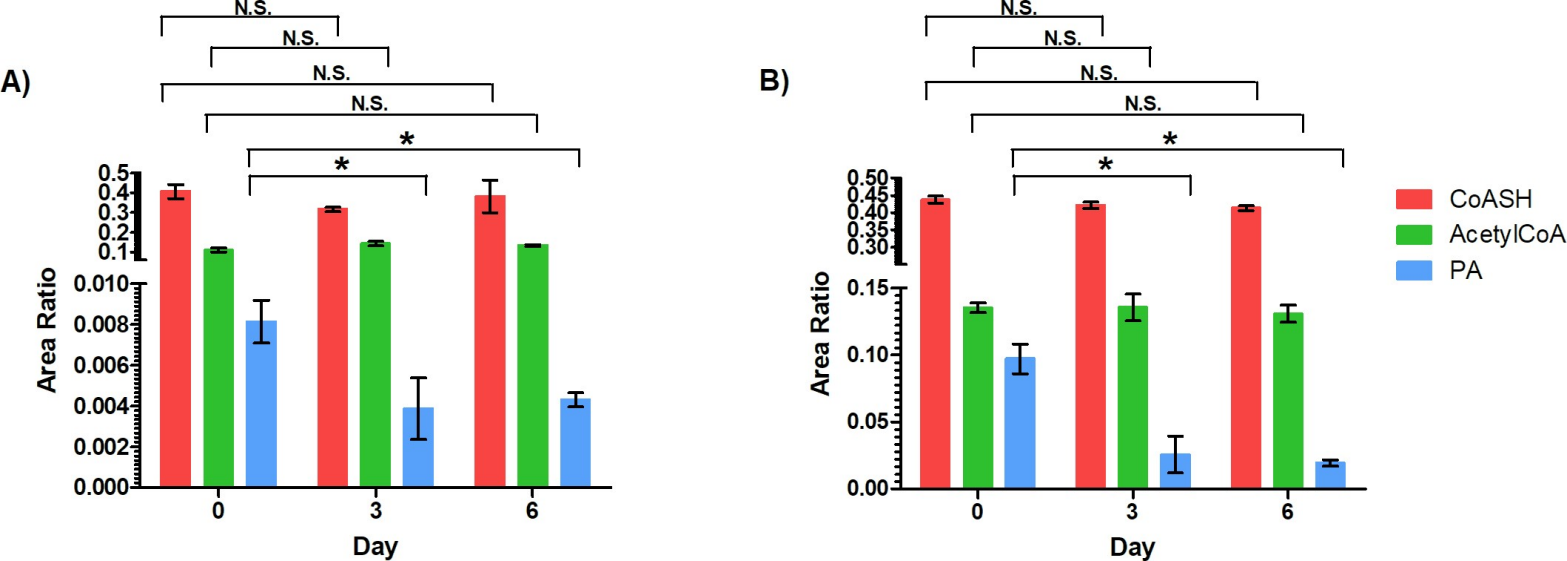
Levels of free CoA (CoASH), acetylCoA and pantothenic acid (PA) in male mouse A) liver and B) brain after administration of a low PA diet for 3 and 6 days. *p < 0.02; N.S. No Significance. Error bar, mean ± SD for triplicate samples.

To determine the half-life of CoASH and acetylCoA in mouse liver and brain, [^13^C_3_-^15^N]-PA was orally administered to the healthy mice. In a preliminary study conducted in male mice, a low PA diet period of three and six days before treatment was evaluated to facilitate the incorporation of [^13^C_3_-^15^N]-PA into CoASH and acetylCoA. Then, levels of endogenous PA, CoASH and acetylCoA were measured. As shown in Fig 1, the PA restriction diet reduced PA levels in liver and brain as early as day 3 while maintaining unaltered CoASH and acetylCoA levels and the health status of the animals. Because of this, 3 days was selected to trigger the incorporation of the labelled PA into CoASH and acetylCoA.

**Fig 2:**
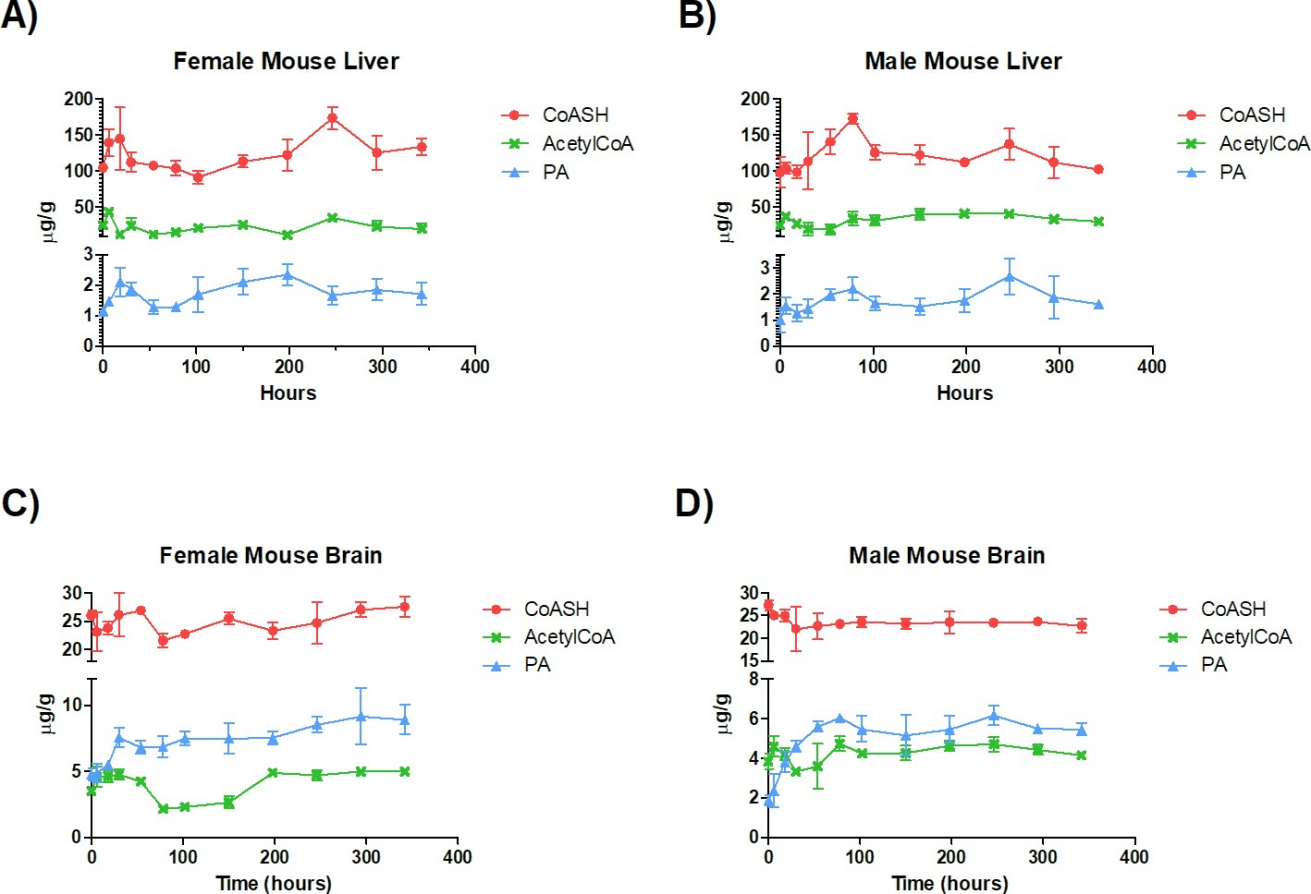
Level of unlabelled pantothenic acid (PA), free CoA (CoASH) and acetylCoA in A) female and B) male mouse liver and C) female and D) male brain after administration of a single oral dose of [^13^C_3_-^15^N]-PA. C57BL/6N mice were kept for 3 days under a PA-free diet and then orally dosed with 25 mg/kg of [^13^C_3_-^15^N]-PA. Liver and brain samples were harvested over a collection period of 0–342 h and unlabelled PA, CoASH and acetylCoA level were measured by LC-MS.

This result is in agreement with a previous study conducted by Shibita K. *et al*. [35], in which the author showed that the administration of a PA-free diet to rats decreased PA levels in a variety of tissues while CoASH and acetylCoA were not affected. Thus, for half-life determination, mice of both genders were kept on a low PA diet for 3 days and a standard rodent diet was established just before administering a single oral dose of [^13^C_3_-^15^N]-PA at 25 mg/kg to reflect physiological conditions during half-life determination. The labelled material was used in the biosynthetic pathway leading to formation of [^13^C_3_-^15^N]-CoASH and [^13^C_3_-^15^N]-acetylCoA (+4 AMU species) that were measured in brain and liver tissues, together with the unlabelled species, by LC-HRMS up to 342 h post-dose. Mice were sacrificed at selected timepoints (three animals/timepoint) and tissues were collected, snap-frozen in dry ice and stored at -80°C until LC-HRMS analysis. The timeframe was selected on the basis of a preliminary experiment performed with just male mice (not shown). The tissue content of unlabelled and labelled PA, CoASH, acetylCoA species was measured using a method able to quantify the endogenous levels and labelled species at C_max_ level. The area ratio of labelled species (peak area analyte/peak area internal standard) was used to determine CoASH and acetylCoA half-lives in tissues. In the latest time points, samples (from 102 to 342 h) labelled metabolites levels had fallen below LOQ. The linearity of response between C_max_ and 10% of C_max_ was thus assessed diluting C_max_ extracts (containing the highest level of labelled species) with blank matrix extracts (containing IS) and plotting the peak area of labelled-analyte (normalized for IS response) *versus* the inverse of the dilution factor. The slope of the linear regression model (y = mx + c) of the dilution-response curve was compared to the slope of the standard curve of the corresponding unlabelled metabolite. Response was considered linear below LOQ if slopes were similar (±20%). As shown in S1 and S2 Figs, a linear response below LOQ (up to 10 folds dilution of C_max_) was obtained, both in the brain and liver, for all the metabolites (CoASH and acetylCoA +4 AMU). All the timepoints collected fell in the linear range and were thus included in the half-life calculation. As shown in Fig 2 and Table 3, levels of unlabelled metabolites in the male and female liver and brain were stable during the entire period of observation. PA instead was minimal at t = 0 as a consequence of the low PA diet and after restoration of a regular diet, reached steady level within 18 and 30 h in the female and male liver, respectively, and 30 and 54 h in the female and male brain, respectively. The PA level, then, remained stable until the end of the study.

**Table 3:** Average endogenous level of pantothenic acid (PA), free CoA (CoASH) and acetylCoA in the male and female C57BL/6N mouse liver and brain.

	Endogenous concentration in liver (μg/g_tissue_)^§^		Endogenous concentration in brain (μg/g_tissue_)^§^	
	Male	Female	Male	Female
PA (n = 3 mice)	1.9 ± 0.6#	1.8 ± 0.4#	5.6 ± 0.6#	7.80 ± 1.2#
	1.0 ± 0.5*	1.2 ± 0.1*	1.9 ± 0.2*	4.7 ± 0.5*
CoASH (n = 36 mice)	119.9 ± 25.7	122.1 ± 26.8	23.8 ± 2.1	24.9 ± 2.6
acetylCoA (n = 36 mice)	31.8 ± 9.0	21.6 ± 10.2	4.2 ± 0.6	4.6 ± 0.5

^§^Means ± standard deviation.

^#^levels upon restoration of a regular diet; n = 3 animals.

*levels after 3 days of low PA diet; n = 3 animals.

**Fig 3:**
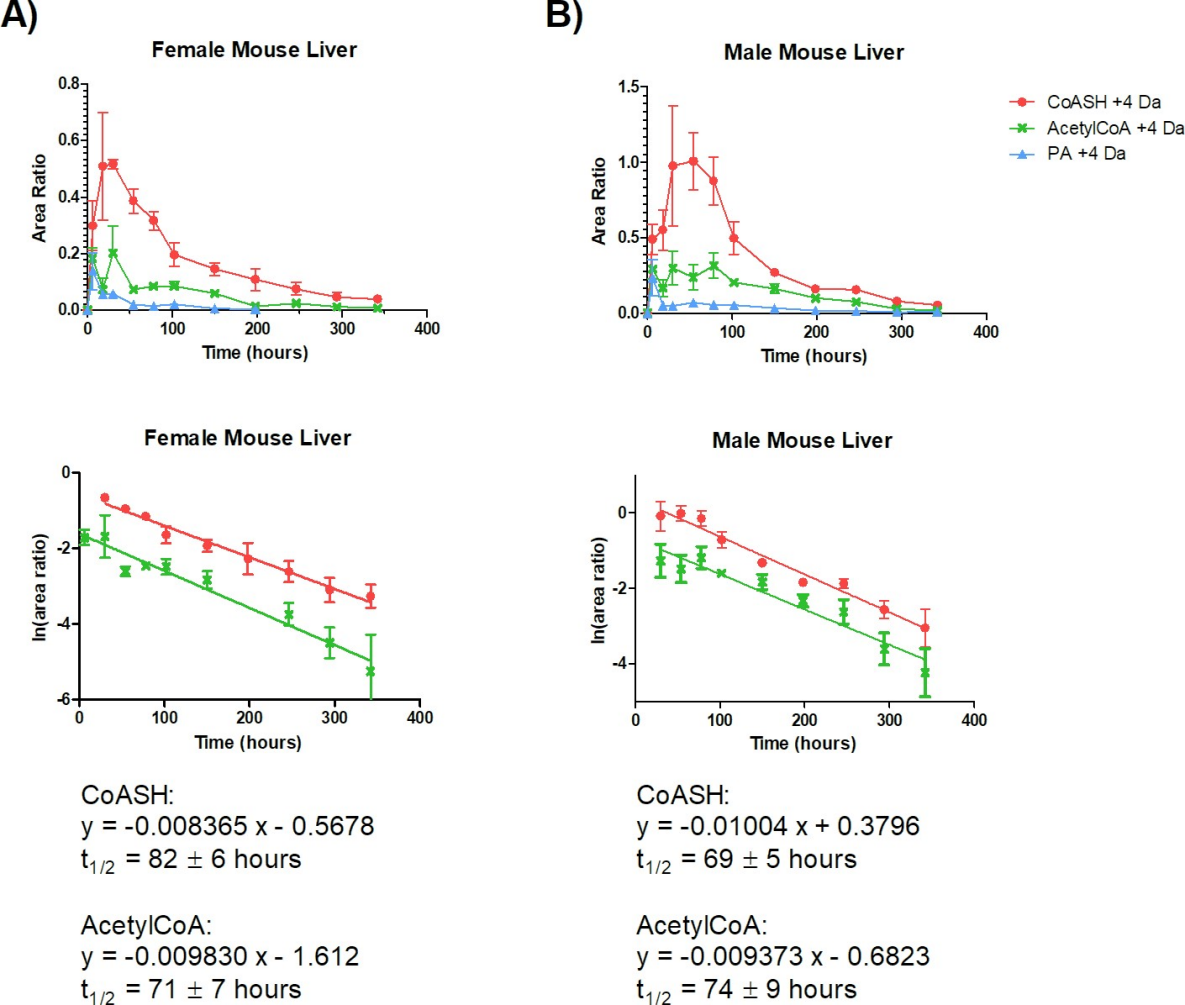
Half-life of free CoA (CoASH) and acetylCoA in A) female B) male mouse liver. C57BL/6N mice were kept for 3 days under a PA-free diet and then orally dosed with 25 mg/kg of [^13^C_3_-^15^N]-PA. Liver samples were collected and analysed by LC-HRMS to determine labelled (+4 AMU) and unlabelled CoASH and acetylCoA levels. The metabolite half-life was calculated from C_max_ to the last time point collected. Error bars, mean ± SD for triplicate samples.

**Fig 4:**
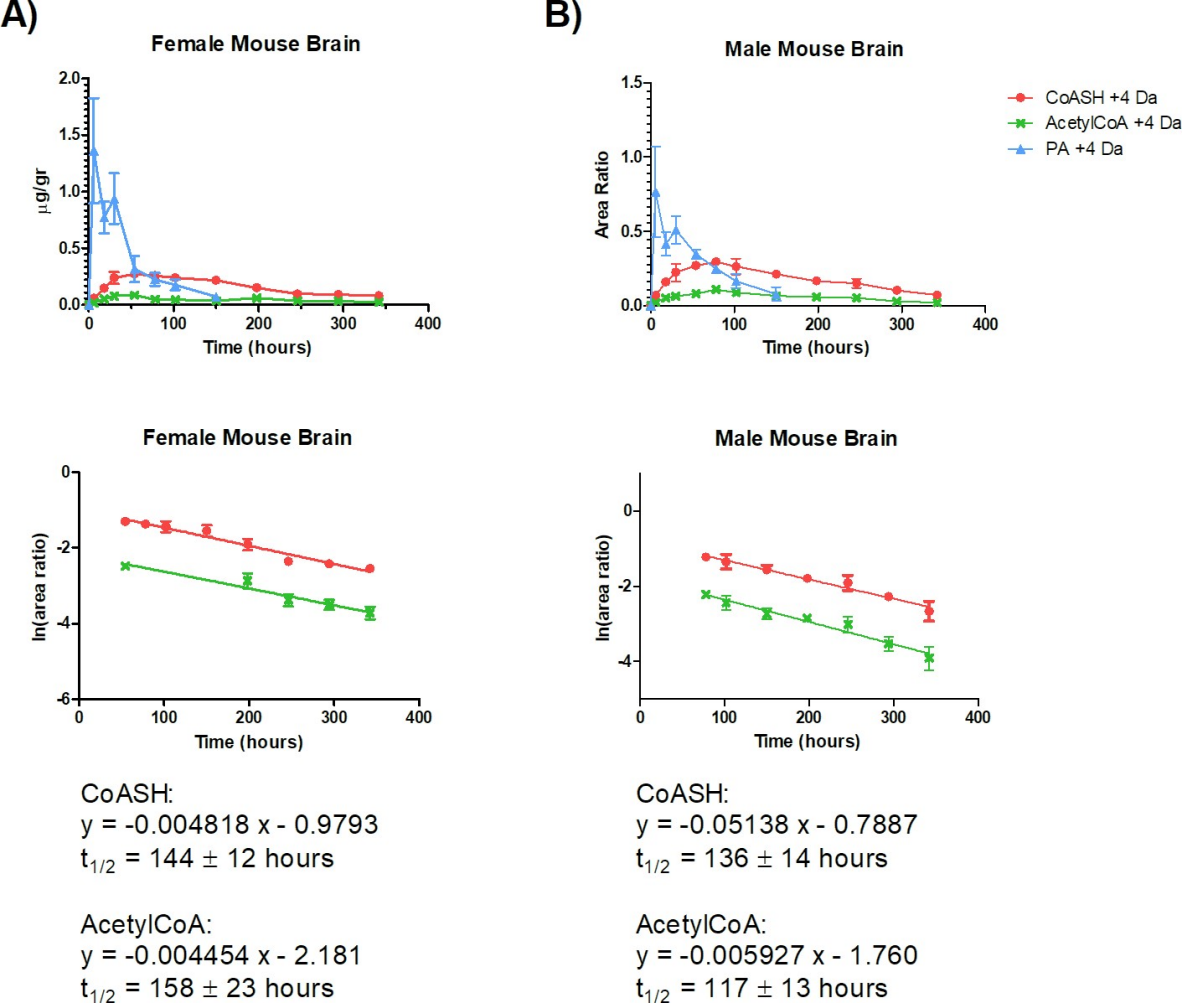
Half-life of free CoA (CoASH) and acetylCoA in A) female B) male mouse brain. C57BL/6N mice were kept for 3 days under a PA-free diet and then orally dosed with 25 mg/kg of [^13^C_3_-^15^N]-PA. Brain samples were collected and analysed by LC-HRMS to determine the labelled (+4 AMU) and unlabelled CoASH and acetylCoA levels. The metabolite half-life was calculated from C_max_ to the last time point collected. Error bars, mean ± SD for triplicate samples.

As shown in Figs 3 and 4, in liver and brain, [^13^C_3_-^15^N]-PA concentration was maximum at 6 h after dosing, which was the first time point collected.

In the liver, [^13^C_3_-^15^N]-PA C_max_ was 1.4 ± 0.8 and 1.8 ± 0.8 μg/g_liver_ in male and female, respectively, the half-life was <18 h in both genders. In the brain, [^13^C_3_-^15^N]-PA C_max_ was 2.3 ± 1.0 and 5.5 ± 2.0 μg/g_brain_ in male and female, respectively, the half-life was 47 ± 9 h in male and 29 ± 4 h in female. [^13^C_3_-^15^N]-PA T_max_ was 6 h (first time point after dosing) for both organs and genders. The shorter half-life of labelled-PA in liver reflects the faster metabolic rate of this tissue when compared to the brain.

CoASH and acetylCoA had a comparable turnover. Their turnover rate was significantly different between the brain and liver, being slower in the former (p < 0.01). No evident gender difference was observed. In particular, CoASH half-life was 69 ± 5 h and 82 ± 6 h in the male and female liver, respectively. The corresponding values for acetylCoA were 74 ± 9 h and 71 ± 7 h in male and female liver, respectively. In the male and female brain, CoASH half-life was 136 ± 14 h and 144 ± 12 h, respectively. The corresponding values for acetylCoA were 117 ± 13 h and 158 ± 23 h in male and female brain, respectively. A summary of the half-life result is reported in Table 4.

**Table 4:** Summary of [^13^C_3_-^15^N]-pantothenic acid ([^13^C_3_-^15^N]-PA), free CoA (CoASH) (+4 AMU) and acetylCoA (+4 AMU) half-life in male and female C57BL/6N mouse brain and liver.

	T_1/2_ (hours)			
	Brain		Liver	
	Male	Female	Male	Female
[^13^C_3_-^15^N]-PA	47 ± 9	29 ± 4	<18 h	<18 h
CoASH (+4 AMU)	136 ± 14	144 ± 12	69 ± 5	82 ± 6
AcetylCoA (+4 AMU)	117 ± 13	158 ± 23	74 ± 9	71 ± 7

### CoASH, acetylCoA and total CoA turnover rate in male mice (intrastriatal study)

**Fig 5:**
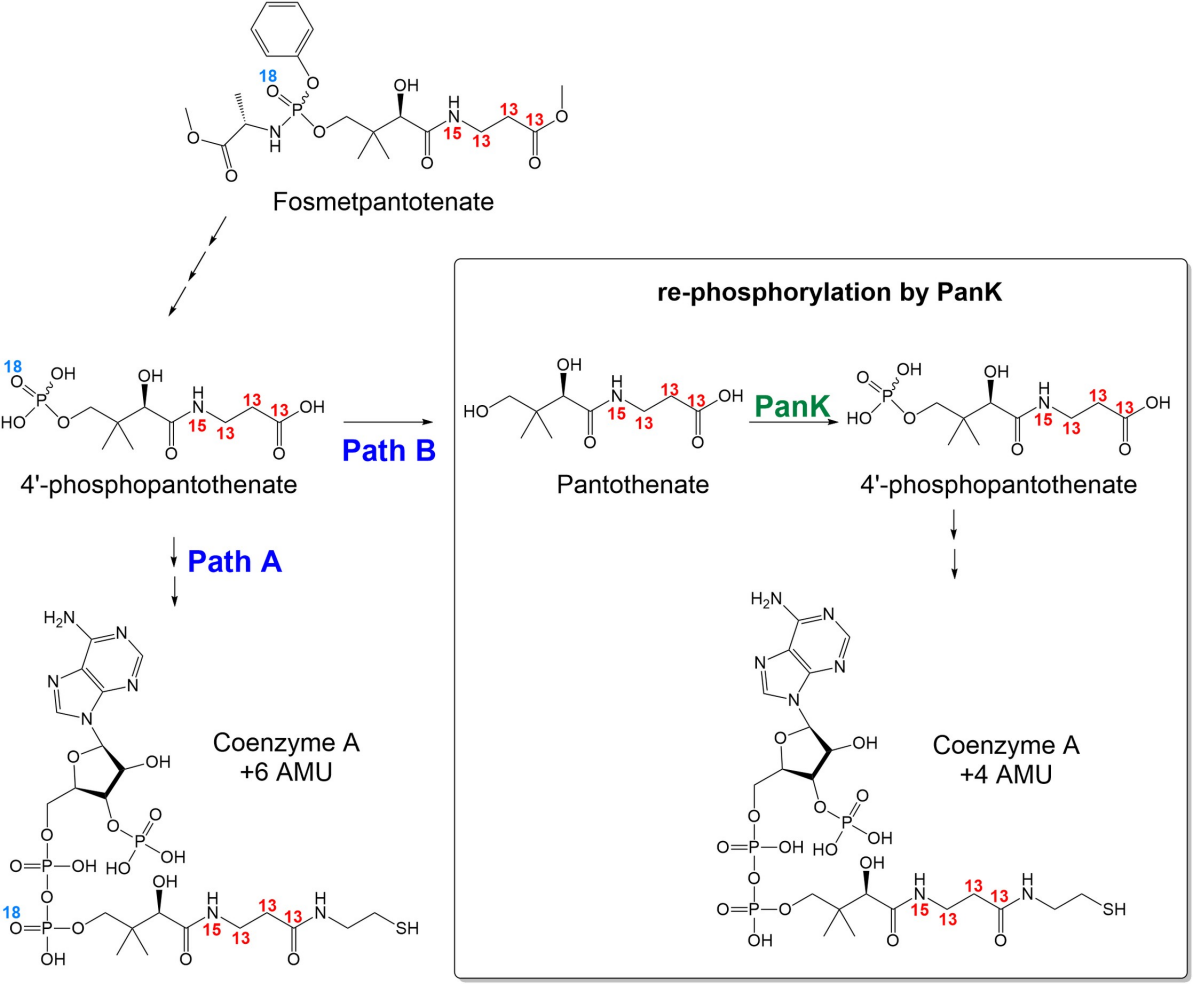
Formation of labelled-CoA species from labelled-fosmetpantotenate (+6 AMU).

An additional study was performed to confirm the half-life results in the brain of mice. This confirmation was performed using only male mice to restrict the use of animals in a surgical procedure of high severity. In this new study, male C57/Bl6 mice (n = 3 per time point) received bilateral intrastriatal injections of 250 μg (125 μg each striatum) of isotopically labelled-fosmetpantotenate (+6 AMU) [31,36]. Fosfometpantotenate a 4’-phosphopanthotenic acid precursor, enters the CoA and acyl-CoA biosynthetic pathways resulting in the formation of +6 and +4 AMU labelled CoA species (Fig 5).

Beside CoASH and acetylCoA, also total CoA was determined to account for all the acyl-CoA species endogenously present in the brain. At selected time points, the brain hemisphere samples were dissected and CoASH, acetylCoA and total CoA (unlabelled, +4 and +6 AMU) levels were measured by LC-MSMS over 336 h. The concentration of the labelled metabolites was used to calculate the turnover rate of CoASH, acetylCoA and total CoA. The use of endogenous metabolites depleted matrix and ion-pair chromatography helped improve the LOQ by 5–10 fold when compared to the standard addition combined with Monolitich/HR-MS method used for the PO study. Thus, the sensitivity was sufficient to measure the labelled metabolites formation and disappearance over the total time period of the experiment.

The labelled species, absent in the pre-dose samples, were already observed at 24 h after dosing and decreased from 24 h (+4 AMU ones) or 96 h (+6 AMU ones) to 336 h. The level of unlabelled CoASH, acetylCoA and total CoA remained stable throughout the entire period of observation (Table 5).

**Table 5:** Average endogenous level of unlabelled free CoA (CoASH), acetylCoA and total CoA in male C57/Bl6 mouse brain (n = 27) during the intrastriatal infusion study over a period of 336 h after dosing.

	Average endogenous concentration (μg/g_brain_)^§^ n = 27 male mice
Total CoA	25.4 ± 4.1
CoASH	19.6 ± 1.7
acetylCoA	1.9 ± 0.3

^§^Means ± standard deviation.

**Fig 6:**
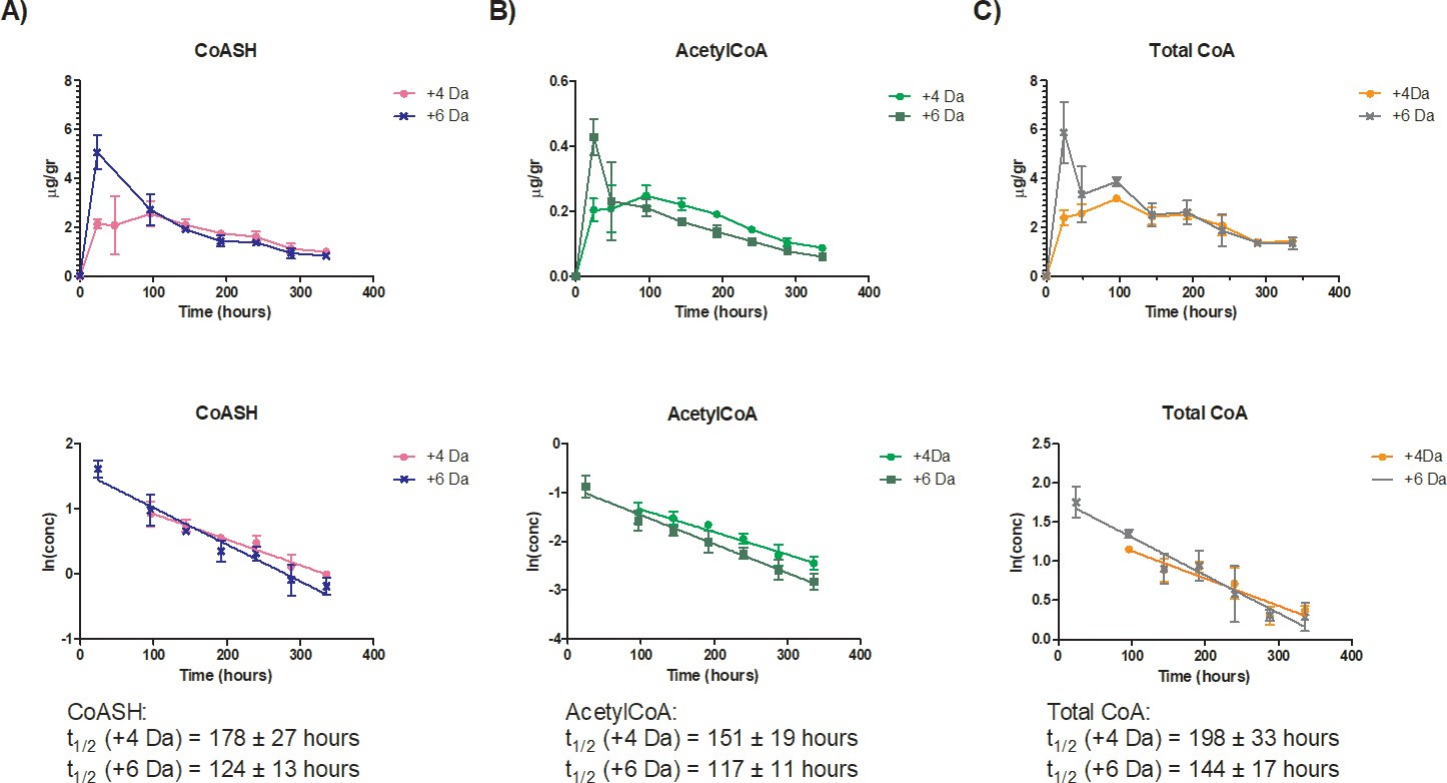
Half-life in male mouse brain of A) free CoA (CoASH) (+4 and+6 AMU), B) acetylCoA (+4 and +6 AMU) and C) total CoA +4 and +6 AMU. C57/Bl6 mice received an intrastriatal injection of 125 μg of +6 AMU labelled-fosmetpantotenate at each brain hemisphere (250 μg total dose) and brain samples were collected over a period of 336 h after dosing. For CoASH +4, acetylCoA +4 and total CoA +4 the time interval 96–336 h was considered for the half-life calculation. For CoASH +6, acetylCoA +6 and total CoA +6 the time interval 24–336 h was used and t = 48 h was excluded from the regression. Error bars, mean ± SD for triplicate samples.

The half-life was (Fig 6): 178 ± 27 h (for CoASH +4 AMU), 124 ± 13 h (for CoASH +6 AMU), 151 ± 19 h (for acetylCoA +4 AMU), 117 ± 11 (for acetylCoA +6 AMU), 198 ± 33 h (for total CoA +4 AMU) and 144 ± 17 h (for total CoA +6 AMU).

The slower turnover rate of the +4 AMU species was probably due to their regeneration from catabolism of the +6 AMU ones (see Fig 5, pathway B and C), as shown by their different T_max_ (24 h and 96 h for + 6 AMU and + 4 AMU species, respectively). ^18^O/^16^O exchange could also contribute to the longer half-life value observed for the +4 AMU CoA species.

A summary of half-life results in mouse brain is reported in Table 6.

**Table 6:** Summary of half-life in mouse brain for free CoA (CoASH), acetylCoA and total CoA (+4 and +6 AMU) after striatal injection of +6 AMU labelled-fosmetpantotenate.

T_1/2_ (hours) in male mouse brain		
	+4 AMU	+6 AMU
CoASH	178 ± 27	124 ± 13
AcetylCoA	151 ± 19	117 ± 11
Total CoA	198 ± 33	144 ± 17

The results obtained after the intrastriatal administration of labelled-fosmetpantotenate are in accordance with the results obtained after the PO dosing of labelled PA.

## Discussion

The knowledge of the *in vivo* turnover rate of the main representative species of CoA is crucial to define the dosing regimen of drugs targeting CoA deficiency. However, despite the relevance of this information, an accurate evaluation of CoA half-life in important tissues like the liver and brain is still lagging behind. We conducted two studies (PO and intrastriatal) to determine PA, CoASH, acetylCoA half-life in the male and female mouse brain and liver and the total CoA in the male mouse brain.

In the PO study, all animals were fed with PA deficient diet for 3 days to favour labelled-PA incorporation into CoA species while maintaining endogenous levels and the health status of the animals. Then, before the oral administration of [^13^C_3_-^15^N]-PA, a standard rodent diet was administered until the end of the study. The decay over time of the biosynthetically formed labelled-CoA species was measured by LC-MS. The obtained results were confirmed by the intrastriatal study, in which the labelled fosmetpantotenate was injected directly into the brain of the male mice. For the two studies, the well-being of the animal was monitored and no adverse effects were observed. In both studies, the endogenous CoA level was unaltered for the duration of the entire experiment, assuring that the animals did not suffer from CoA deprivation, nor did they experience metabolic impairment.

In the PO study, we observed a very long half-life in the brain for CoASH (136 ± 14 h (male) and 144 ± 12 (female)) and acetylCoA (117 ± 13 h (male) and 158 ± 23 h (female)) and in the liver (CoASH 69 ± 5 h (male) and 82 ± 6 h (female), actylCoA 74 ± 9 h (male) and 71 ± 7 h (female)). The turnover was shorter in the liver than in the brain, reflecting organ function and physiology. No significant gender differences were observed.

Similar results were confirmed in the intrastriatal study conducted in male mice only; 124 ± 13 h for CoASH, 117 ± 11 h for acetylCoA and 144 ± 17 h for total CoA.

To the best of our knowledge, this is the first report of *in vivo* CoA turnover rate in the mouse brain. An estimation of CoASH half-life in the mouse liver was published by Zhang *et al*. [37]. These authors investigated the biochemical and genetic alterations occurring in mice when inhibiting CoA biosynthesis using a pantothenate structural analogue, hopantenate (HoPan), which inhibits all active PANK isoforms [38]. HoPan, administered to male and female mice (100 μg/g/day by gavage) depleted the free and total CoA in mouse tissues. The drop of CoA levels was particularly evident in the liver and the kidney suggesting a faster turnover rate for CoASH in these tissues compared to the brain and heart, which is in agreement with our results. In the HoPan treated mice, the CoA half-life in the liver was estimated as 20–24 h in males and about 90 h in females. While the turnover rate in female mice was in agreement with our result (82 ± 6 h), the result in the male mice was noticeably shorter (20–24 h compared to 69 ± 5 h). This discrepancy might be due to the different health status of the male and female animals after the HoPan treatment (the former expiring after 5 days while the latter surviving for 16 days from the beginning of the treatment) and compared to our study in which the animals were healthy for the entire period of observation and no adverse effects were recorded. In the HoPan study, a severe liver impairment was also observed.

Our data illustrate for the first time the turnover rate of CoA in the mouse brain and liver in physiological healthy conditions, showing different tissue turnover rates.

## Conclusions

In this study we reported results for the first *in vivo* turnover rate determination of CoASH, acetylCoA and total CoA in the brain and liver of healthy mice. These results demonstrate that all CoA species investigated have a long turnover rate, shorter in the liver than in the brain (about 3 days in liver and 5–6 days in brain). The difference between the two organs was not surprising and probably reflects the faster metabolic activity of the liver. Gender differences, not observed in the PO study, where not investigated in the intrastriatal study which was conducted only in male mice. The turnover rate of the CoA species could be useful to develop a therapeutic agent aimed to increase CoA level in deficiency conditions leading to serious neurodegenerative disease like PKAN.

## Supporting information

S1 Fig Linearity of CoASH (+4 AMU) MS response below LOQ. Comparison of CoASH (+4 AMU) curves obtained by dilution (2, 5 and 10 folds) of the C_max_ sample extract with blank A) liver and B) brain mouse matrix extracts with curves of the unlabelled standard matabolites in the same matrix. The slope of the labelled and unlabelled curves was compared. Similar slope (±20%) indicated linearity below LOQ.
(TIF)

S2 Fig Linearity of acetylCoA (+4 AMU) MS response below LOQ. Comparison of acetylCoA (+4 AMU) curves obtained by dilution (2, 5 and 10 folds) of the C_max_ sample extract with blank A) liver and B) brain mouse matrix extracts with curves of the unlabelled standard matabolites in the same matrix. The slope of the labelled and unlabelled curves was compared. Similar slope (±20%) indicated linearity below LOQ.
(TIF)

## Abbreviations

AGC: Automatic gain control
ACN: Acetonitrile
AcylCoA: Esterified coenzyme A
AMU: Atomic mass unit
C_max_: Concentration at maximunm
CNS: Central nervous systhem
CE: Collision energy
CoA: Coenzyme A
CoASH: Free CoA
COASY: CoA synthase
CS: Calibration standard
CXP: Collision cell exit potential
DMSO: Dimethyl sulfoxide
DP: declustering potential
DPCK: Dephospho-CoA kinase
EP: Entrance potential
FA: Formic acid
FWHM: Full width half maximum
HoPan: Hopantenate
IS: Internal standard
KOH: Potassium hydroxide
LC-MS: Liquid chromatography mass spectrometry
LC-HRMS: Liquid chromatography high resolution mass spectrometry
LC-MSMS: Liquid chromatography tandem mass spectrometry
LOQ: limit of quantification
MeOH: Methanol
MRM: multiple reaction monitoring
PA: Pantothenic acid
PANK: Pantothenate kinase
PANK2: Pantothenate kinase 2
PANK3: Pantothenate kinase 3
PKAN: Pantothenate kinase-associated neurodegeneration
PO: Per os
PPCDC: (*R*)-4’-phospho-N-pantothenoylcysteine decarboxylase
PPAT: 4’-phosphopantetheine adenylyltransferase
QC: Quality control
TFA: Trifluoroacetic acid
tSIM: Targeted selected ion monitoring
