# Supplementary figures and images for: Turnover rate of coenzyme A in mouse brain and liver
Source: PLoS One. 2021 May 21;16(5):e0251981. doi: 10.1371/journal.pone.0251981 (PMC8139499; doi:10.1371/journal.pone.0251981)

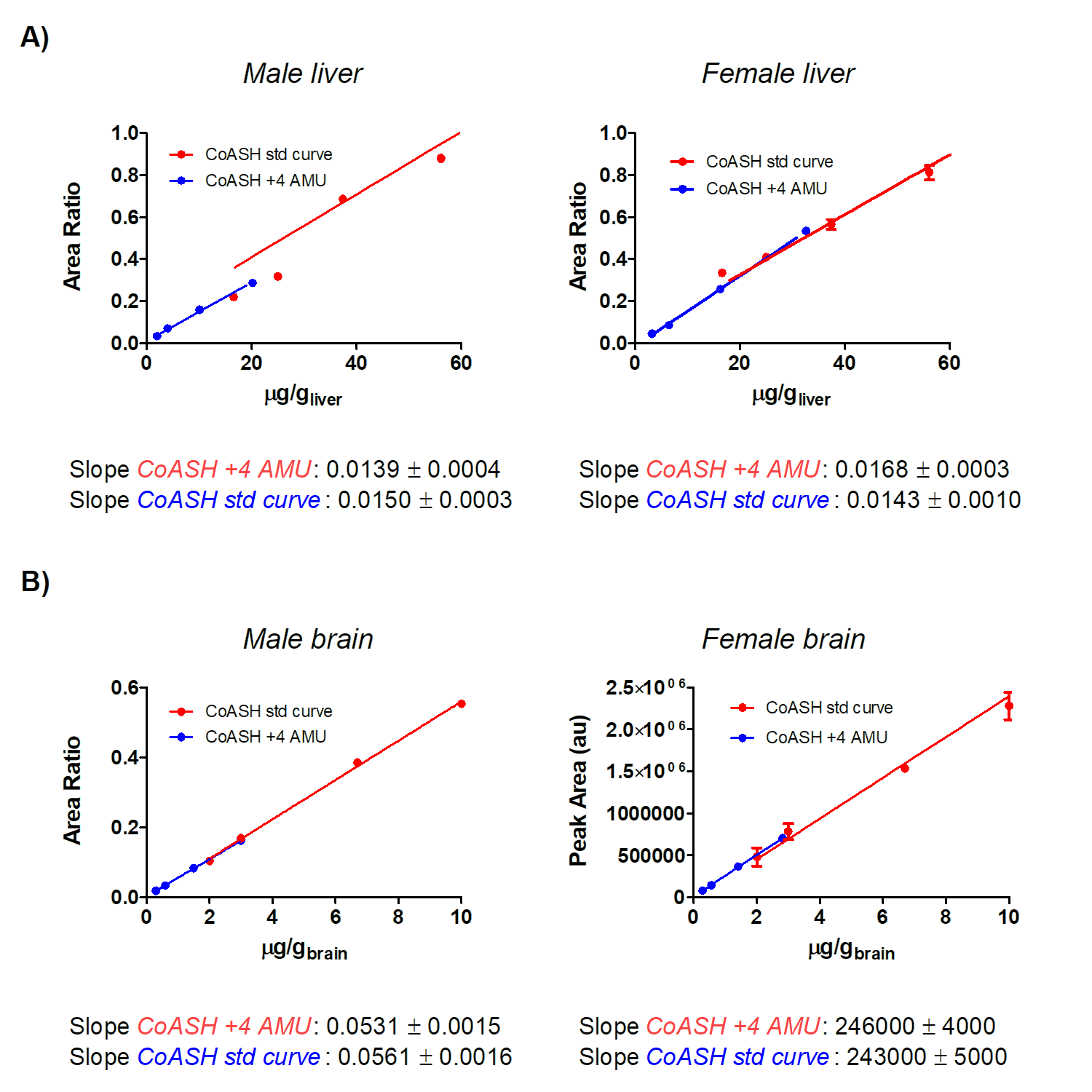

Supplement: S1 Fig — Comparison of CoASH (+4 AMU) curves obtained by dilution (2, 5 and 10 folds) of the Cmax sample extract with blank A) liver and B) brain mouse matrix extracts with curves of the unlabelled standard matabolites in the same matrix. The slope of the labelled and unlabelled curves was compared. Similar slope (±20%) indicated linearity below LOQ. (TIF) [file pone.0251981.s001.tif]

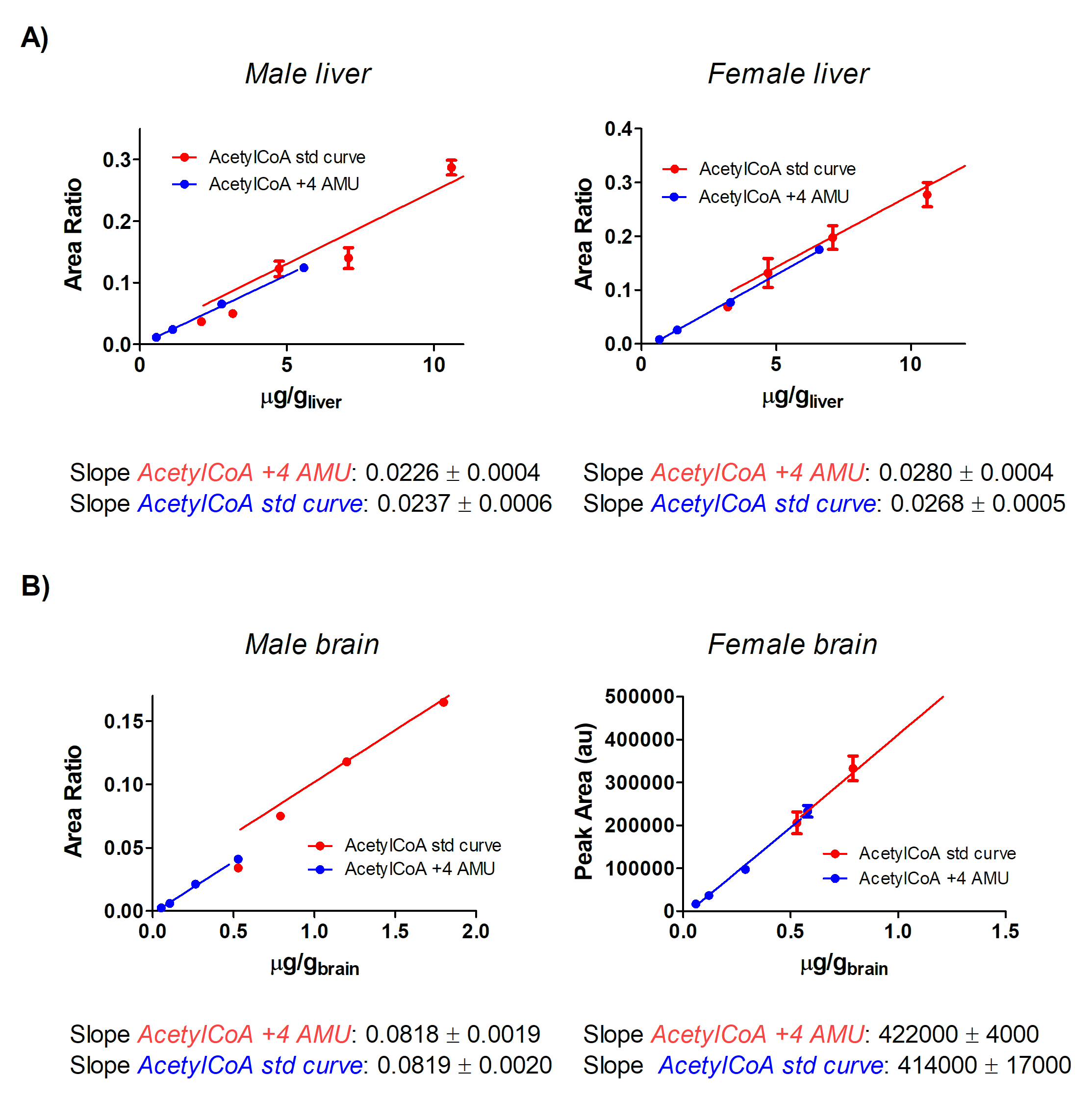

Supplement: S2 Fig — Comparison of acetylCoA (+4 AMU) curves obtained by dilution (2, 5 and 10 folds) of the Cmax sample extract with blank A) liver and B) brain mouse matrix extracts with curves of the unlabelled standard matabolites in the same matrix. The slope of the labelled and unlabelled curves was compared. Similar slope (±20%) indicated linearity below LOQ. (TIF) [file pone.0251981.s002.tif]
